# A chromatin code for alternative splicing involving a putative association between CTCF and HP1α proteins

**DOI:** 10.1186/s12915-015-0141-5

**Published:** 2015-05-02

**Authors:** Eneritz Agirre, Nicolás Bellora, Mariano Alló, Amadís Pagès, Paola Bertucci, Alberto R Kornblihtt, Eduardo Eyras

**Affiliations:** Universitat Pompeu Fabra, E08003 Barcelona, Spain; IFIBYNE-UBA-CONICET, Departamento de Fisiología, Biología Molecular y Celular, Facultad de Ciencias Exactas y Naturales, Universidad de Buenos Aires, (C1428EHA), Buenos Aires, Argentina; Centre for Genomic Regulation, E08003 Barcelona, Spain; Catalan Institution of Research and Advanced Studies (ICREA), E08010 Barcelona, Spain; Present address: Institute of Human Genetics, CNRS UPR 1142, Montpellier, France; Present address: INIBIOMA, CONICET-UNComahue, Bariloche, Río Negro Argentina; Present address: European Molecular Biology Laboratory, Meyerhofstrasse 1, 69117 Heidelberg, Germany

**Keywords:** Chromatin, Splicing, Histones, Splicing code

## Abstract

**Background:**

Alternative splicing is primarily controlled by the activity of splicing factors and by the elongation of the RNA polymerase II (RNAPII). Recent experiments have suggested a new complex network of splicing regulation involving chromatin, transcription and multiple protein factors. In particular, the CCCTC-binding factor (CTCF), the Argonaute protein AGO1, and members of the heterochromatin protein 1 (HP1) family have been implicated in the regulation of splicing associated with chromatin and the elongation of RNAPII. These results raise the question of whether these proteins may associate at the chromatin level to modulate alternative splicing.

**Results:**

Using chromatin immunoprecipitation sequencing (ChIP-Seq) data for CTCF, AGO1, HP1α, H3K27me3, H3K9me2, H3K36me3, RNAPII, total H3 and 5metC and alternative splicing arrays from two cell lines, we have analyzed the combinatorial code of their binding to chromatin in relation to the alternative splicing patterns between two cell lines, MCF7 and MCF10. Using Machine Learning techniques, we identified the changes in chromatin signals that are most significantly associated with splicing regulation between these two cell lines. Moreover, we have built a map of the chromatin signals on the pre-mRNA, that is, a chromatin-based RNA-map, which can explain 606 (68.55%) of the regulated events between MCF7 and MCF10. This chromatin code involves the presence of HP1α, CTCF, AGO1, RNAPII and histone marks around regulated exons and can differentiate patterns of skipping and inclusion. Additionally, we found a significant association of HP1α and CTCF activities around the regulated exons and a putative DNA binding site for HP1α.

**Conclusions:**

Our results show that a considerable number of alternative splicing events could have a chromatin-dependent regulation involving the association of HP1α and CTCF near regulated exons. Additionally, we find further evidence for the involvement of HP1α and AGO1 in chromatin-related splicing regulation.

**Electronic supplementary material:**

The online version of this article (doi:10.1186/s12915-015-0141-5) contains supplementary material, which is available to authorized users.

## Background

Alternative splicing is a key mechanism to generate functional diversity in most eukaryotic cells. Its importance is underlined by the fact that it potentially affects more than 90% of human genes [1,2] and its deregulation is frequently associated with severe diseases [3]. The regulation of alternative splicing has been generally thought of as being primarily controlled by the activity of splicing factors and by the elongation rate of the RNA polymerase II [4]. However, during the last few years it has become clear that regulation of pre-mRNA splicing is more complex than initially thought and a new picture has emerged whereby various mechanisms of regulation are coupled in a network of interactions between RNA, chromatin and protein factors [5-7]. The analysis of nucleosome positioning data has suggested a general role of chromatin in exon definition [8-10], and a number of experiments have provided evidence that chromatin can interact with splicing through various ways [11-23]. One of these mechanisms involves adaptor proteins that can bridge between modified histones and splicing factors [11-14]. In a different mechanism, spliceosomal factors can influence the chromatin state and affect transcriptional activity [15-18].

A third, non-mutually exclusive, mechanism proposes that changes in chromatin that interfere with RNA polymerase II (RNAPII) elongation can also affect splicing regulation [19-23]. In this context, the CCCTC-binding factor (CTCF), which is implicated in diverse functions related to the global organization of chromatin [24] and acts as a barrier for the spreading of heterochromatin [25], has been shown to be capable of stalling elongation of RNAPII [26] and to regulate the splicing of upstream exons with weak splice sites [23]. Similarly, siRNAs directed by the Argonaute protein (AGO1) to intragenic regions induce chromatin changes that alter RNAPII elongation, thereby affecting splicing [21] in a mechanism analogous to transcriptional gene silencing [27,28]. Although the main function of Argonaute proteins is traditionally described to be performed in the cytoplasm in relation to the post-transcriptional gene silencing mechanism [29], there is increasing evidence for a nuclear role [27,28,30-37]. In this regard, we have recently shown by ChIP-Seq that AGO1 binds to active transcriptional enhancers in mammalian cells and that through this binding it regulates the constitutive and alternative splicing of neighboring genes [37]. Interestingly, the *Drosophila* Argonaute protein Ago-2 associates with CTCF at promoters [33], and its binding sites include part of the CTCF motif [33,36]. On the other hand, there is also evidence that members of the heterochromatin protein 1 (HP1) family have a general role in co-transcriptional RNA processing and splicing [22,38,39]. HP1 is a family of non-histone proteins that recognize methylated H3K9, are responsible for the establishment and maintenance of heterochromatin, and associate with other non-histone proteins [40-42]. Furthermore, HP1 proteins have been implicated in heterochromatin formation linked to AGO1 activity in alternative splicing [21,35]. These results raise two interesting questions: whether the CTCF, AGO1 and HP1 proteins associate together at the chromatin level in human cells, and whether these associations play any role in alternative splicing regulation.

To address these questions, we analyzed the combinatorial code of AGO1, CTCF, and HP1α together with RNAPII activity as well as the histone marks H3K27me3, H3K9me2, H3K36me3, and total H3 and 5metC signals in relation to the alternative splicing differences between two cell lines, a non-tumorigenic immortalized breast epithelial cell line, MCF10, and its cancer-derived counterpart, MCF7. Using Machine Learning (ML) techniques, we uncovered the chromatin signals that associate significantly with the splicing regulation of the pre-mRNA comparing these two cell lines, which leads us to describe a *chromatin-based RNA-map* that explains nearly 70% of all the regulated alternative splicing events between the two cell lines. Moreover, we find a significant association between HP1α and CTCF in relation to the alternative splicing events and a putative binding motif for HP1α.

## Results and discussion

### Association of chromatin signals with alternative splicing events

**Figure 1:**
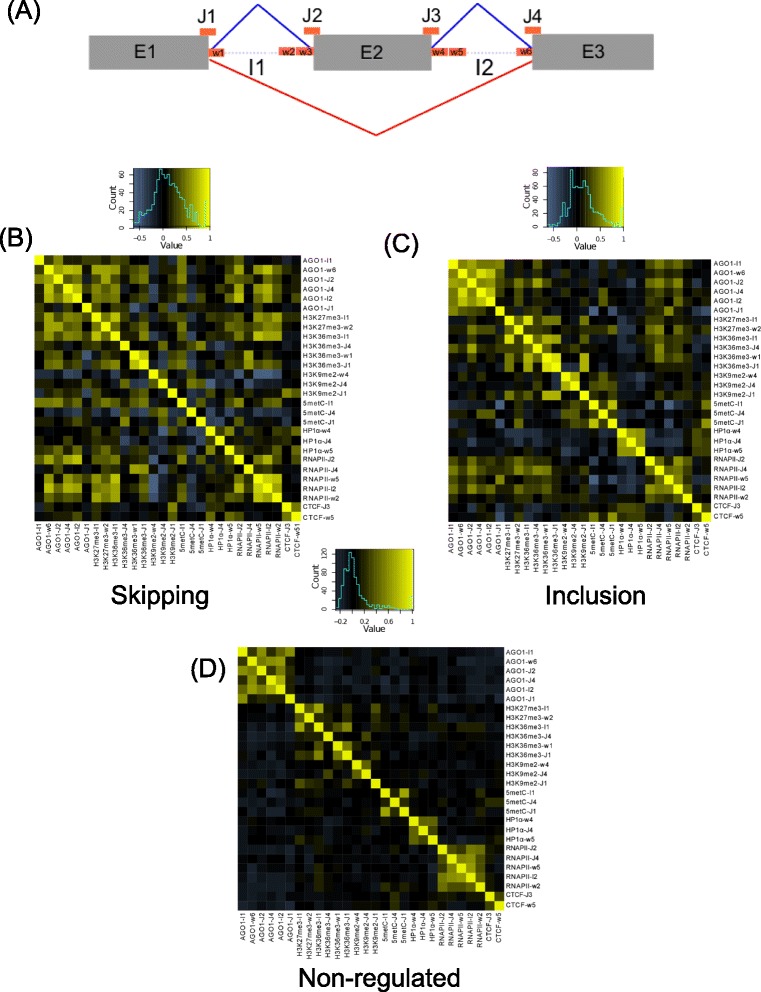
Attributes and their correlations. **(A)** Diagram of the 15 windows defined on exon cassette events: 300 nt length windows flanking exons (w1,…, w6), 200 nt length regions covering 100 nt on either side of the exon boundaries (J1,…,J4), the entire exons (E1, E2 and E3) and the extent of the flanking introns (I1 and I2). Pearson correlation coefficients were calculated pairwise for the different attributes in skipping **(B)**, inclusion **(C)** and non-regulated **(D)** events. The three heatmaps show in the same order those attribute pairs that have R ≥0.6 or R ≤−0.5 in inclusion and/or skipping events, and involving attributes from two different experiments. Correlation coefficient values are given in Additional file 1: Tables S1–S5. The heatmaps for the pairwise correlations for all the 120 attributes for inclusion, skipping and non-regulated events are shown in Additional file 1: Figure S1.

We used a splicing microarray analysis to obtain alternative splicing events (ASEs) showing a significant change between MCF7 and MCF10. From this group we selected an equal number of inclusion and skipping events (442) located in genes showing no change in expression (Methods). These ASEs will be referred to as regulated events. On the other hand, we used ChIP-Seq data for AGO1, CTCF, H3K27me3, H3K9me2, H3K36me3, RNAPII, HP1α, total H3 and 5metC in the same cell lines, and considered those read-clusters with a significant signal with respect to Control ChIP-Seq experiments (*P*-value <0.05) (Methods). For each ASE, we defined 15 different windows (Figure 1A), and for each window, we calculated the relative enrichment of the read densities between MCF7 and MCF10 for each one of the ChIP-Seq experiments. This defined one attribute for each sample-window pair, with a value corresponding to the relative enrichment z-score of the ChIP-Seq signal. We thus generated 8 x 15 = 120 attribute values for each alternative splicing event (Figure 1A).

Pairwise correlation analysis of these 120 attributes for skipping, inclusion and non-regulated events showed significant differences between regulated and non-regulated events (Figures 1B to D) (Additional file 1: Figure S1), with higher correlation between attributes in regulated events compared to non-regulated events. Besides the high correlations between attributes corresponding to the same ChIP-Seq experiment in neighboring regions, we observed that the strongest correlations involved the intronic regions flanking the regulated exons. In particular, we found that H3K27me3-I1 versus H3K9me2-I1 and H3K36me3-I1 versus H3K27me3-I1 have high correlation in the three groups of events, indicating that these signals would not differentiate regulated from non-regulated events (Additional file 1: Tables S1, S2 and S3). On the other hand, there were differences in other attributes, which could potentially separate inclusion and skipping events (Additional file 1: Tables S4 and S5). Regulated events for which inclusion is upregulated in MCF7 cells compared to MCF10 cells (inclusion events) showed high correlations between histone marks on the exon-intron junctions (Figure 1B) (Pearson correlation R = 0.85 for H3K36me3-J1 versus H3K9me2-J1, R = 0.85 for H3K36me3-J3 versus H3K9me2-J1), between histone marks and AGO1 on the first upstream exon-intron junction and on the second downstream exon-intron junction (R = 0.62 for H3K36me3-J4 versus AGO1-J2 and AGO1-J4) and on the first exon-intron junction and downstream windows (R = 0.61 for 5metC-J1 versus CTCF-w5) (Additional file 1: Table S2). In contrast, in regulated events that show skipping in MCF7 (skipping events) we found mostly high correlations between AGO1 and RNAPII (R = 0.74 for AGO1-I2 versus RNAPII-J2, R = 0.73 for AGO1-w6 versus RNAPII-I2), CTCF and histone modifications (R = 0.63 for CTCF-J3 versus H3K9me2-w2 and CTCF-w1 versus H3K27me3-w5 R = 0.67) and between AGO1 and H3K27me3 downstream of the alternative exon (R = 0.63 for AGO1-w6 versus H3K27me3-I1) (Figure 1C). We also found anti-correlating attributes for inclusion and skipping events: inclusion events showed mainly AGO1 anti-correlating with histone marks and DNA methylation (R = −0.63 for AGO1-I1 versus 5metC-J1, R = −0.52 for AGO1-w2 versus H3K9me2-w5) and HP1 anti-correlating with H3K27me3 (Pearson correlation factor R = −0.58 for HP1-w1 versus H3K27me3-J1). Similarly, skipping events also showed various anti-correlation patterns (R = −0.68 for RNAPII-w4 versus H3K9me2-w4 and R = −0.64 for RNAPII-w3 versus H3K27me3-w6). In contrast, most of these correlations did not appear in non-regulated events. The correlations and anti-correlations found in regulated events and the differences with non-regulated events suggest a cooperative role of the different chromatin signals and factors in relation to specific patterns of alternative splicing regulation.

### A chromatin code of splicing regulation

**Figure 2:**
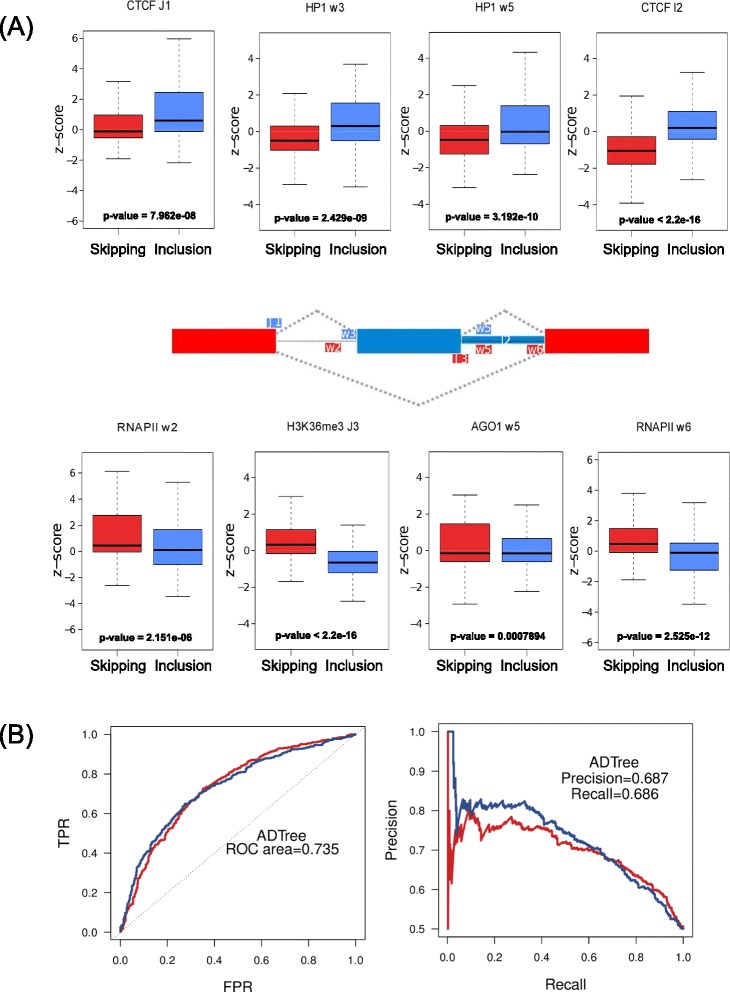
Chromatin-based RNA-map. **(A)** Each boxplot represents the relative change in signal densities as z-score values correlated with inclusion or skipping exons for the selected attributes, separated according to whether they show enrichment in skipping exons (red) or in inclusion exons (blue). The plots show the Kolmogorov-Smirnov test *P*-value for the comparisons of the distributions for each attribute. The exon triplet diagram in the middle shows the regions of the selected attributes (Additional file 1: Table S6). **(B)** Receiver operating characteristic (ROC) curves and precision-recall curves for the ADTree model, separated for inclusion (blue) and skipping exons (red). We indicate the average area under the ROC curve (0.735), precision (0.687) and recall (0.686) for both classes from the 10-fold cross-validation of the model (Additional file 1: Table S7). ADTree, Alternate Decision tree.

In order to test a possible association of chromatin signals with the changes in splicing patterns, we built a binary classification model to separate inclusion from skipping events, selecting the predictors from the 120 attributes described above (Methods). To select the most informative attributes for the classification, we used a combination of feature selection methods (Methods) (Additional file 1: Table S6). This resulted in 15 attributes, involving seven of the ChIP-Seq signals considered (Additional file 1: Figure S2), which best separate inclusion and skipping events. Among the most informative attributes, we found HP1α and CTCF downstream of the alternative exon in relation to inclusion events; and AGO1, H3K36me3 and RNAPII in relation to skipping events. These attributes show significant relative differences in chromatin signal densities (z-scores) between exon inclusion and skipping (Figure 2A). In particular, we found that AGO1 in the downstream window (w5) associates with the direction of the splicing change, that is, splicing events with an increase of AGO1 signal between MCF7 and MCF10 downstream of the alternative exon were more frequently associated with skipping (Figure 2A). We also found an increase of HP1α in the downstream and upstream regions associated with inclusion (Figure 2A). The downstream window (w5) showed a different pattern in association with skipping for the AGO1 signal and in association with inclusion for the HP1α signal (Figure 2A). Similarly, an increase of CTCF on the downstream intron (I2) was related to inclusion (Figure 2A). On the other hand, for H3K36me3 and RNAPII we found the opposite pattern: an increase on the flanking regions of the regulated exon correlated with skipping (Figure 2A). In contrast, the relative enrichments of these signals in a set of non-regulated exons are centered on zero and distributed between inclusion and skipping events (Additional file 1: Figure S2). Although 5metC was selected as informative by the feature selection procedures (Methods), the signal density around the regulated events and non-regulated events did not show clear differences (Additional file 1: Figure S2). Using the 15 most informative attributes (Figure 2A), we used cross-validation with an Alternate Decision tree (ADTree) classifier to obtain 606 (68.552%) correctly labeled events (282 inclusion, 324 skipping) (Additional file 1: Table S7 and Additional file 2) from the original 884 events (receiver operating characteristic (ROC) area 0.735, precision 0.687, recall 0.686) (Figure 2B). Moreover, using only the top features related to the CTCF, HP1α and AGO1 signals we recover about 58% of the events correctly (ROC area 0.6, precision = 0.58, recall = 0.58) (Additional file 1: Table S7). Furthermore, features related to the total H3 signal did not provide any predictive value and normalization of the histone modifications by total H3 did not affect the results (Methods). Our results indicate that a considerable number of alternative splicing events can be explained by the differences in the relative enrichment of histone marks and the factors CTCF, HP1α and AGO1 (Additional file 1: Figure S2).

**Figure 3:**
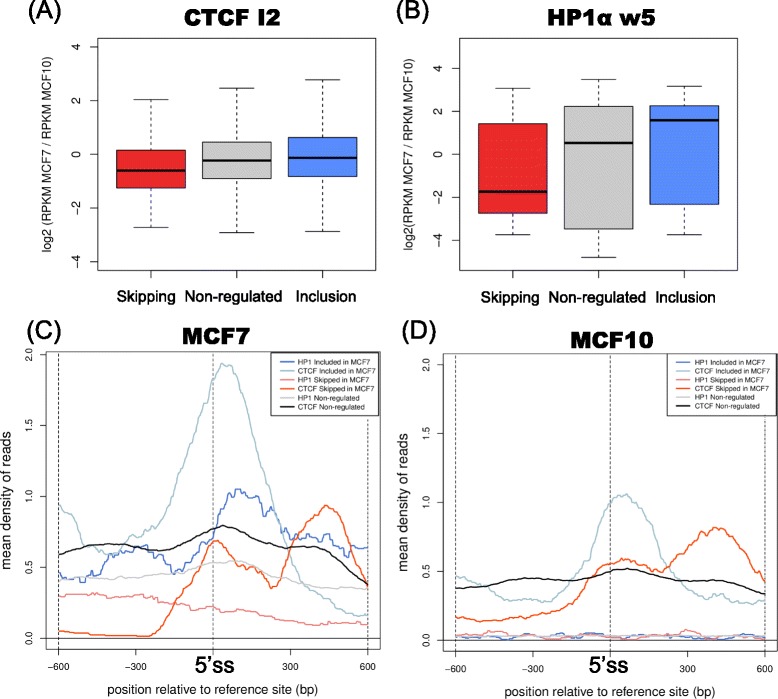
Read profiles on regulated events. Density differences between MCF7 and MCF10, measured as the log_2_-ratio of RPKM values (y-axis) in **(A)** the I2 region for CTCF in skipped exons (red), inclusion exons (blue) and non-regulated exons (gray) (Kolmogorov-Smirnov test *P*-value <0.01 for all comparisons); and in **(B)** the region w5 for HP1α, in skipped exons (red), inclusion exons (blue) and non-regulated exons (gray) (Kolmogorov-Smirnov test *P*-value <0.01 for all comparisons). Profiles for HP1α and CTCF in MCF7 **(C)** and MCF10 **(D)** around 5′ splice-sites. The profiles show the mean read densities (y-axis) from -600 bp to 600 bp (x-axis) centered at the 5′ss of the regulated alternative exon (E2) for both ChIP-Seq samples, separately for skipped exons (red), included exons (blue) and non-regulated exons (gray). ChIP-Seq, chromatin immunoprecipitation-sequencing; CTCF, CCCTC-binding factor; HP1, heterochromatin protein 1.

To provide a different view of the chromatin signals in relation to splicing regulation, we calculated the read density properties for the most informative ChIP-Seq features, HP1α and CTCF downstream of regulated exons. In the region I2, CTCF has significant density enrichment in included exons and significant depletion in skipped exons in MCF7 relative to MCF10, compared with non-regulated exons (Figure 3A). HP1α shows a similar enrichment and depletion pattern in the region w5 (Figure 3B) but not as discriminating as with the enrichment attributes (Figure 2A). We calculated the profiles for the CTCF and HP1α read densities for regulated exons correctly classified as skipped and included by the model, first removing those exons that overlap with the first or second exon in some transcript of the gene to avoid biases from the transcription start site (TSS). In MCF7, CTCF shows a higher density downstream of inclusion events compared with skipping events (Figure 3C). In contrast, in MCF10 CTCF is present at similar levels downstream of inclusion and skipped events, although at a lower density than for MCF7 in inclusion events (Figure 3D). For HP1α in MCF7 we observed a higher density downstream of included exons compared with skipped exons (Figure 3C and Additional file 1: Figure S3A), whereas it is depleted in MCF10 downstream of regulated exons (Figure 3D and Additional file 1: Figure S3). The patterns for inclusion events are in agreement with the proposed splicing code that predicts that an increase of CTCF or HP1α binding downstream of exons is linked to increased inclusion (Figure 2A). On the other hand, skipping events showed a lack of co-localization of CTCF and HP1α, suggesting that the splicing modulation by CTCF, RNAPII and histone marks may be strongly influenced by the presence of HP1α (Additional file 1: Figure S3). The most relevant feature involving RNAPII corresponds to the region w6, close to the downstream exon (Figure 2). However, RNAPII tends to localize close to regulated exons, more prominently in regions where CTCF and HP1α are present and in the direction of inclusion (Additional file 1: Figure S3). In contrast, even though AGO1 was selected as a relevant attribute in the downstream region (w5), we did not find much difference in the AGO1 read densities between skipping and inclusion (data not shown). However, comparison of the normalized densities over the w5 region showed a higher signal of AGO1 in MCF7 in the direction of skipping (Additional file 1: Figure S4), consistent with our previous findings [37]. The fact that AGO1 appears as a selected feature in the w5 like HP1α but in the opposite direction of regulation and with a weaker signal suggests that AGO1 and HP1α may antagonize for a limited number of events.

### A possible association of CTCF, HP1α and AGO1 in splicing regulation

**Figure 4:**
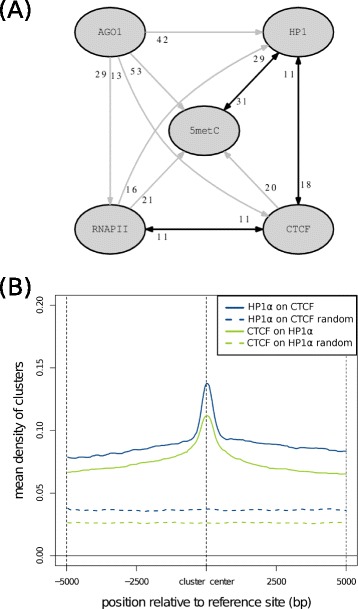
Association between HP1α and CTCF binding sites. **(A)** Graph of significant genome-wide associations between AGO1, CTCF, HP1α, RNAPII and 5metC binding sites. The black double arrows indicate the significant associations between HP1α and CTCF, HP1α and 5metC and CTCF and RNAPII, whereas the directional gray arrows indicate the significant one-sided associations (Additional file 1: Table S8). The number beside each arrow indicates the proportion of clusters (rounded to the closest integer) that overlap with the sites of the factors connected by the arrow. **(B)** Mean densities of HP1α clusters centered at CTCF clusters (blue line), compared with randomized HP1α clusters (dashed blue line); and mean densities of CTCF clusters (green line) centered at the HP1α clusters, compared with randomized CTCF clusters (dashed green line). Randomized clusters were calculated by relocating each cluster in an arbitrary new position in the same chromosome, avoiding satellites, gaps, pericentromeric regions and the overlap with any other random cluster. AGO1, argonaute 1 protein; CTCF, CCCTC-binding factor; HP1, heterochromatin protein 1; RNAPII, RNA polymerase II; 5metC, 5-methycytosine.

Our results indicate a possible association between the analyzed proteins in chromatin-mediated regulation of splicing. We thus decided to further investigate this by calculating the significance of the overlap between their binding signals (Methods). Interestingly, we found that there was a positive genome wide association between CTCF and HP1α, CTCF and RNAPII and 5metC and HP1α (Additional file 1: Table S8). In contrast, we did not find any association of 5metC with CTCF, which agrees with their proposed antagonistic activity [23,43]. Although AGO1 was not found to be strongly associated with any other signal, we found that 41.8% of AGO1 clusters were overlapping with HP1α, 52.6% with 5metC and 13.1% with CTCF, whereas only 1.9%, 3.4% and 0.8% of these signals, respectively, were associated with AGO1 clusters, indicating that AGO1 may be associated specifically with CTCF and HP1α but not the other way around (Figure 4A) (Additional file 1: Table S8). Furthermore, the association of CTCF and HP1α is recapitulated at intragenic regions (CTCF with HP1α z-score = 90.791 and HP1α with CTCF z-score = 5.826). This is also supported by the overlap analysis of clusters. We observed that CTCF clusters tend to localize at HP1α cluster positions and vice versa (Figure 4B). This co-localization is recapitulated at intragenic regions (Additional file 1: Figure S5A). We also found an association between CTCF and RNAPII downstream of the included exons (regions w4 and w5) (CTCF localizing with RNAPII z-score = 10.20 and RNAPII localizing with CTCF z-score = 3.246), consistent with a CTCF mediated accumulation of RNAPII [23].

### Candidate DNA binding motifs for HP1α and AGO1

**Figure 5:**
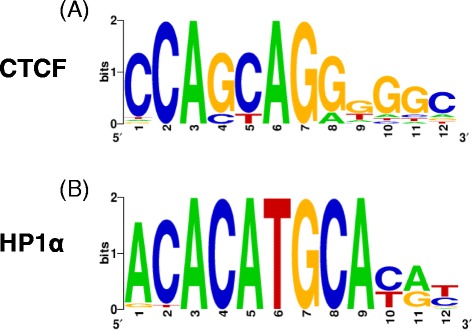
Consensus motifs for CTCF **(A)** and HP1α **(B)**. Motifs were built using the top 10 heptamers from significant CTCF and HP1α clusters (Methods). CTCF, CCCTC-binding factor; HP1, heterochromatin protein 1.

Although there is evidence for the association of AGO1 and HP1α with chromatin [37,41], specific candidate DNA binding motifs have not been proposed before. The strong overlap of these two factors with CTCF binding sites raises the question whether AGO1 and HP1α may have specific binding motifs. Our datasets provide a good opportunity to test this hypothesis. We thus calculated enriched DNA motifs in the AGO1, CTCF and HP1α cluster sets independently (Methods). Due to the high overlap between the three samples, we assessed the enrichment of heptamers using all significant non-overlapping clusters, and built consensus motifs represented as position weight matrices (PWMs) using both complementary strands (Methods). As a validation of our approach, we confirmed that the motif found for CTCF (Figure 5A) coincided with the one previously reported [44]. For HP1α clusters we obtained an AC-rich motif (Figure 5B), whereas for AGO1 we obtained a motif with the conserved hexamer AGGTCA (Additional file 1: Figure S5B). Since HP1 proteins bind methylated H3K9 [40-42], as a further check for the HP1α motif we decided to test whether H3K9me2 significant clusters would give rise to a similar motif. We applied the same method and found for H3K9me2 a short palindromic motif that matches the consensus CWGCWG (Additional file 1: Figure S5C). Interestingly, this motif includes CAGCAG and its reverse complement, which partly matches the CTCF motif (Figure 5A) and shows a strong overlap with CTCF clusters (23.5%), but not with HP1α (1.7%) or AGO1 (4.6%) clusters. To further determine how specific the motifs are, we calculated the density profiles of the motifs over each set of significant ChIP-Seq clusters. The profile of HP1α motif over the significant clusters indicates that this motif is very specific (Additional file 1: Figure S6A). The motif recovered for CTCF is highly enriched in CTCF clusters but also over HP1α clusters (Additional file 1: Figure S6B). In contrast, AGO1 motif shows very little specificity, most likely due to the fact that there are few AGO1 clusters that do not overlap with either HP1α or CTCF (Additional file 1: Figure S6C). We further tested whether HP1α or any of the motifs found were particularly enriched over H3K9me3 significant clusters. The CTCF motif shows the highest density over H3K9me2 clusters; whereas HP1α shows the lowest density (Additional file 1: Figure S7). These analyses suggest that the found binding motif for HP1α may indeed be associated with its binding to DNA.

## Conclusions

In this work, we have derived a chromatin code for splicing that involves binding signals for HP1α and CTCF, as well as AGO1, RNAPII and histone marks, activity around regulated exons. Feature selection and cross-validation shows that this regulatory code is predictive for nearly 70% of the alternative splicing events regulated between two cell lines, MCF7 and MCF10, providing further evidence for a role of chromatin in the regulation of alternative splicing. This code also provides evidence for specific associations of various factors in relation to splicing differences between the two studied cell lines. Our model shows that AGO1 activity downstream of alternative exons correlates with splicing changes in the direction of skipping in MCF7 compared to MCF10, providing further indication that AGO1 association with chromatin could be implicated in splicing regulation [4,21,35,37]. We also recovered the previously described increased binding of CTCF binding downstream of inclusion events [23]. Additionally, the density of RNAPII downstream of regulated exons, which tends to co-occur with CTCF and HP1α, is an informative attribute to predict splicing change; and a relative increase in the region flanking the exon correlates with exon skipping in MCF7 compared to MCF10. The association of the RNAPII density related to exon definition has been observed before [45] and there is much evidence supporting a regulation of alternative splicing associated with RNAPII elongation rates [19,46,47]. Our results corroborate the importance of RNAPII occupancy in the exon inclusion or skipping, and provide directionality in the relation between density changes and the pattern of differential splicing between cell lines.

H3K36me3 also appeared as a relevant mark for splicing decisions in our model. Several reports have described H3K36me3 as an exon marker [8-10,48] and there is evidence of higher densities of H3K36me3 at constitutive exons compared to alternative exons [8,49]. However, the opposite pattern has also been described, as for specific genes an increased density of H3K36me3 has been related to exon skipping [5,20,50], which agrees with our code. Since we only analyzed splicing events in genes that do not change expression, our results imply that the observed changes in H3K36me3 signal near exon boundaries were not a consequence of gene expression, and could indeed correspond to a role in splicing.

Interestingly, we found a strong association between CTCF and HP1α signals genome-wide and intragenically, and the activity of both factors correlated with exon inclusion. Besides acting as insulator, CTCF is involved in the splicing regulation of some exons as an antagonist of DNA methylation [23] and also works as a barrier for spreading of heterochromatin [24,25], through which it can influence RNAPII elongation [26]. Our analyses show that HP1α binding downstream of the cassette exons, with the co-localization of CTCF, affects alternative splicing. HP1α belongs to a family of non-histone chromosomal proteins and is a key player in the transcriptional gene silencing (TGS) pathway [21]. HP1 proteins have already been linked before to the regulation of splicing by chromatin [21,22,35,39]. In particular, a study published at the time we had concluded this work also describes a positional effect on splicing for HP1 proteins, providing further evidence of the relevance of the HP1 family in linking chromatin with RNA processing [39] and giving support to our model. The same study found that HP1 proteins could act as mediators between DNA methylation and splicing for a subset of the regulated events [39]. Although there have been previous reports of a relation between DNA methylation and alternative splicing [39,51,52], we did not find it to be a strong determinant of the splicing changes between MCF7 and MCF10 cells, indicating that the HP1-dependent code that we describe is related to a DNA-methylation independent effect that may be more prevalent in the investigated cell types.

Even though there is only limited evidence of direct DNA-binding for HP1α [41], we found a consensus motif associated with the significant HP1α ChIP-Seq signals, which is highly specific to the significant HP1α ChIP-Seq signals and non-overlapping with the motifs for CTCF, AGO1 or H3K9me2. HP1 proteins generally consist of two highly conserved domains. While one of the domains is known to bind H3K9me, the other one acts as the interaction interface with other proteins [42]. The two domains are separated by a hinge region of variable length, which has been related to DNA and RNA binding [42,53]. The found motif may be related to a sequence-specific interaction of this protein region with DNA, which may act as a modulator of the interaction of HP1 with H3K9 methylation. Recent analyses also provide evidence of HP1 proteins interacting with RNA binding proteins [39], highlighting their plasticity and central role in RNA processing regulation linked to chromatin.

We also found a frequent overlap of AGO1 with CTCF and HP1α clusters, but not the other way around. Moreover, we found HP1α in the same downstream region as AGO1 but in the direction of inclusion, and regulating a distinct set of events. Depletion of AGO1 expression can induce splicing changes in both directions but generally decreases splicing efficiency [37]. Our analyses show that AGO1 and the co-localized CTCF and HP1α produce splicing changes in opposite directions. Despite the co-localization of AGO1 with CTCF and HP1α binding sites, we found a weak but independent binding motif for AGO1. Recent analyses have produced candidate binding motifs for *Drosophila* [33,36] and mouse [54] Argonaute proteins. However, our motif does not resemble any of these motifs, suggesting a DNA-independent association of AGO1 with chromatin [36,37].

Different models to predict the splicing outcome, also called splicing codes, have been proposed before [55,56], but these did not include chromatin marks or proteins that interact with chromatin, such as HP1, AGO1, CTCF and RNAPII, as described here. Our analyses thus complement these previous descriptions by incorporating these new determinants of alternative splicing regulation. Although motifs in the pre-mRNA sequence remain the main determinants of splicing regulation [56], our analysis indicates that a considerable fraction may be influenced by the properties of chromatin. There have been previous attempts to establish a general relation between histone marks and splicing regulation [57-60]. However, a predictive model was proposed in only one case [57]. Additionally, these approaches analyzed the relation between chromatin and splicing, looking at one single condition at the time, rather than comparing two conditions, and exons were classified as constitutive or alternative based on RNA data from one single condition, rather than distinguishing those that are regulated from the non-regulated ones between two conditions. Our approach has the advantage that, by comparing two conditions locally, it circumvents the caveats of comparing genomic regions with different sequence and structural properties. Moreover, our approach relates changes of the chromatin signal between two conditions to the splicing changes of exons between the same two conditions, which provides a better descriptor of the association between chromatin changes and splicing regulation.

In summary, we have shown that a chromatin code for splicing can be defined involving HP1α, CTCF, RNAPII, various histone marks and AGO1, which can differentiate patterns of skipping, inclusion and non-regulated exons between two conditions. Additionally, the conserved motif found for HP1α and the presence of HP1α and AGO1 in the described splicing code provides further support for their involvement in chromatin-related splicing regulation.

## Methods

### Datasets

Splicing changes between MCF7 and MCF10 were measured using a splice junction array (Affymetrix HJAY) (data available at GSE38864). We considered 1,694 regulated cassette events with significant change (average probe fold-change >1.5) in the comparison between MCF7 and MCF10. Only events from genes that do not change expression significantly (log_2_-fold change *P*-value >0.01) and do not overlap with non-regulated events (see below) were kept, producing 1,134 events, 442 for inclusion and 692 for skipping. Further, in order to have a balanced training set, we randomly chose 442 events of skipping; hence 442 exon cassette events of each type (inclusion or skipping in MCF7, compared to MCF10) were finally selected. As a control, a set of non-regulated events was selected, defined to be exon triplets tested on the array from the same host genes as the regulated events, such that they do not overlap with regulated events and are negative for splicing regulation according to the array experiments. This resulted in 1,970 non-regulated events.

ChIP-Seq data for AGO1, total H3, H3K36me3, H3K9me2, H3K27me3, HP1α, 5 methylated cytosine (5metC) and RNAPII in MCF7 and MCF10 cells were obtained from GSE56826. For AGO1, we only used data for the 4B8 antibody for both MCF7 and MCF10 cells [37]. As control samples we used ChIP-Seq for a non-specific antibody (immunoglobulin G (IgG)) and specific controls samples for HP1α and 5metC in MCF7 and MCF10 cells (available from GSE56826). Additional ChIP-Seq data for RNAPII in MCF7 cells was obtained from [61] and ChIP-Seq data for CTCF in MCF7 and MCF10 cells was obtained from [62].

Reads were mapped to the reference genome hg18 using Bowtie [63] keeping the best unique matches with at most two mismatches to the reference (−v 2 –best –strata -m 1). Based on the mean size of the fragments obtained after sonication for each sample, mapped reads were extended to 200 nt in the 5′ to 3′ direction using Pyicoteo [64,65], except for AGO1 reads, which were extended to 350 nt. Using BedTools [66] we removed reads overlapping centromeres, gaps, satellites, pericentromeric regions and the ‘Duke excluded’ regions, which are regions of low mappability defined by ENCODE [67]. For each sample, we built clusters with the reads that were overlapping each other on the genomic coordinates, discarding single-read clusters, using Pyicoteo.

In order to avoid the usage of clusters that are possibly part of the background signal and not of the real ChIP-Seq signal, we used the control samples to identify significant clusters. As the coverage of reads between samples and control can be highly variable, in order to estimate the background level, we considered that each sample is composed of a number of regions with high coverage, likely to be significant, and a large number of regions with low coverage, assumed to be equivalent to the background [68,69]. Thus, the clusters overlapping in sample and control were considered, and the number of reads *n* for each cluster was measured. The average enrichment between sample and control in regions with few reads was then used to define the ChIP to control normalization factor (CNF):

$$ CNF=\frac{1}{N}{\displaystyle \sum_{i=1}^Nlo{g}_2\left(\frac{n_i^{(s)}}{n_i^{(c)}}\right)} $$

where ${n}_i^{(s)}$ and ${n}_i^{(c)}$ are the number of reads in sample and control, respectively, over each one of the N regions of overlap between sample and control with <10 reads selected to determine the background. The CNF was then used to calculate a normalized Bayesian *P*-value to estimate the significance of the number of reads in the sample cluster, defined as the conditional probability inferred from control number of reads *n*^(*c*)^ in a given region given a number of sample reads, *n*^(*s*)^ in the same region [70]:

$$ P\left(\left.{n}^{(c)}\right|{n}^{(s)}\right)={(CNF)}^{n^{(c)}}\frac{\left({n}^{(c)}+{n}^{(s)}\right)!}{n^{(c)}!{n}^{(s)}!{\left(1+CNF\right)}^{n^{(c)}+{n}^{(s)}+1}} $$

For each sample, in order to get the maximum number of significant clusters, we selected the clusters below a different *P*-value cut-off: AGO1 and H3K9me2 (*P*-value <0.05), H3K27me3, H3 and H3K36me3 (*P*-value <0.01), HP1α (*P*-value <0.001), 5metC (*P*-value <1e-3), RNAPII (*P*-value <1e-3), CTCF (*P*-value <1e-3). For subsequent analyses, only reads overlapping these significant clusters were used. The final number of significant clusters used for the calculations in MCF-7 (MCF-10) is 14,206 (25,098) AGO1 clusters, 232,089 (346,477) CTCF clusters and 407,133 (436,248) HP1α clusters.

To build the predictive model, 15 regions around each cassette event were considered, consistently with Ensembl annotation (version 54, assembly hg18) [71]. For each region and sample, the read density was calculated using the RPKM (reads per kilobase per million of mapped reads) [72], and the relative enrichment z-score was calculated using Pyicoteo [64]. To test possible effects of nucleosome positioning, all histone modifications were normalized by total H3 signal, but no differences were observed in the results with or without this normalization. The described model does not include this normalization.

Pairwise correlation heatmaps were obtained by calculating Pearson correlation between z-scores for every pair of attributes for each subset of triplets: with E2 either included, skipped or non-regulated, according to the splicing array. The processed datasets are available in a publicly available Biomart [73] instance at [74], where the information for each event is linked to an Ensembl gene and transcript IDs to facilitate the cross queries with Ensembl databases. Datasets can be exported in various formats, including ARFF (attribute-relation file format), which can be uploaded directly into the WEKA system [75] for Machine Learning analyses.

### Accuracy testing and attribute selection

Three attribute selection methods were applied: Information Gain (IG), Correlation Feature Selection (CFS) and Wrapper Subset Evaluator (WSE). IG is defined as the expected reduction in entropy caused by partitioning the examples according to one attribute [76], thus the higher the IG value, the better the attribute can separate skipping and inclusion classes. On the other hand, CFS works by testing the correlation of attributes against the class values (inclusion and skipping) and removing those that have high redundancy (high correlation) between them [77]. In WSE, subsets of attributes are tested iteratively using a 10-fold cross validation and the space of all possible subsets is explored heuristically, such that only those subsets that perform above an optimal threshold are scored as informative [78]. Thus, the WSE method gives the frequency at which each attribute is selected in the optimization procedure. For WSE we used a Genetic Search algorithm to explore different combinations of attributes and an ADTree [79] to evaluate the attributes. Repeated runs of WSE did not change the resulting top attributes. Attributes selected by WSE and CFS at frequencies ≥50% and a position in the IG ranking in the top 50% were finally selected. When the selected attributes corresponded to the same ChIP-Seq signal in overlapping regions, the one with the highest IG was selected. In this way, a minimal set of non-redundant attributes with optimal performance was selected (Additional file 1: Table S6).

For each of the models the accuracy was calculated using 10-fold cross validation. In this procedure, the datasets are split into training and testing sets in 10 different ways. Testing sets were chosen such that each event is predicted just once. The accuracy was measured as the average value of the sensitivity and specificity over all 10 splits. We also reported the number of events, either skipped or included, that were correctly predicted by the model. Since our attributes were expected to be dependent, we applied two different classifiers that were based on dependencies to build the model and test the predictive power of our attributes: a Bayesian Network (BN) [80] and an ADTree [81]. A BN consists of the combination of conditional probabilities between attributes to define a network, where each attribute has a probability distribution given by the conditional probability on one or more parent attributes. ADTree is a classification method based on binary decision trees, using a voting system to combine the output of individual tree models. Each individual model has a tree structure, where each node of the tree represents a binary partition. At every partition a test is performed for every attribute and the test set that maximizes the entropy-based gain ratio [78] is selected, leading to a tree where every leaf contains instances from one class when there is no over-fitting. Individual trees are combined into a single tree using a voting system to weight the contribution from the multiple binary tests into a final classification, which is represented in the leaves. The ADTree has been shown before to be a good learning algorithm for genetic regulatory response [81]. Each model was built with a given number of attributes, for our initial model 120 attributes were used (BN model area under the ROC curve = 0.67 and ADTree model area under the ROC curve = 0.661). The final model was built only with the 15 selected attributes, with an accuracy for the BN model of area under the ROC (AUC) = 0.71, recall = 0.671, precision = 0.673; and for the ADTree model of AUC = 0.735, recall = 0.686, precision = 0.687. Based on the results the ADTree model was selected.

### Cluster association and motif analysis

To study the significance of the co-occurrence of the different ChIP-Seq clusters in specific regions we used the block bootstrap and segmentation method developed in the Encode project [82]. Using a list of genomic regions and two lists of features mapped to them, this method provides a z-score corresponding to the number of standard deviations of the observed overlap compared to the random expected overlap. We ran version 0.8.1 of the script Block Bootstrap and Segmentation method with parameters -r 0.1 -n 10,000, where r is the fraction of each region in each sample and n is the number of bootstrap samples used. As input for this method, ChIP-Seq clusters and mappable genome regions were used.

The motif analysis was carried out in the following way. Given a sample set *S* of *N* sequences and a control set *S*^*(0)*^ of *N*^*(0)*^ sequences, the number of times *n*_*i*,*a*_ that each 7-mer *a* appeared in each sequence *i* was calculated. Likewise, for the control set the number of occurrences ${n}_{i,a}^{(0)}$ of each 7-mer *a* in each sequence *i*, was also calculated. The expected density ${d}_a^{(0)}$ of each 7-mer *a* was then calculated as the ratio between the total number of occurrences in the control set over the total sequence length of the control set:

$$ {d}_a^{(0)}=\frac{{\displaystyle \sum_{i\in {S}^{(0)}}{n}_{i,a}^{(0)}}}{{\displaystyle \sum_{i\in {S}^{(0)}}{l^{(0)}}_i}}, $$

where ${l}_i^{(0)}$ is the length of each sequence in the control set. For each sequence *i* in the sample set and each 7-mer *a*, it was then recorded whether the observed 7-mer count, *n*_*i,a*_, is greater than the expected count, ${d}_a^{(0)}{l}_i$:

$$ \begin{array}{l}{d}_{i,a}=1\kern1em \mathrm{if}\kern0.5em {n}_{i,a}>{d}_a^{(0)}{l}_i\\ {}{d}_{i,a}=0\kern1em \mathrm{otherwise}\end{array} $$

Similarly, for the counts in the control set:

$$ \begin{array}{l}{d}_{i,a}^{(0)}=1\kern1em \mathrm{if}\kern0.5em {n}_{i,a}^{(0)}>{d}_a^{(0)}{l^{(0)}}_i\\ {}{d}_{i,a}^{(0)}=0\kern1em \mathrm{otherwise}\end{array} $$

The sum of the *d*_*i,a*_ values over the sequences *i* represent the number of sequences for which the 7-mer *a* has an observed count greater than expected. Thus, for each 7-mer, the odds-ratio (7-mer score) and corresponding *P*-value were obtained by performing a Fisher test (one-tailed) with these sums for the sample and control sets (Table 1).

**Table 1:** **Contingency matrix for the enrichment analysis of a 7-mer** ***a***

**Dataset**	**More than expected**	**Less than expected**
*S (sample)*	${\displaystyle \sum_{i\in S}{d}_{i,a}}$	$N-{\displaystyle \sum_{i\in S}{d}_{i,a}}$
*S* ^*(0)*^ *(control)*	${\displaystyle \sum_{i\in {S}^{(0)}}{d^{(0)}}_{i,a}}$	${N}^{(0)}-{\displaystyle \sum_{i\in {S}^{(0)}}{d^{(0)}}_{i,a}}$

This motif analysis was carried out independently for CTCF, AGO1 and HP1α clusters. In order to build the consensus motifs, a procedure similar to [83] was carried out. First, the 200 bp sequence centered at the middle position of the cluster was extracted. Only clusters with at least two significant 7-mers were kept, resulting in 401 regions for AGO1, 4,219 for CTCF and 573 for HP1α. A sequence logo and a position frequency matrix (PFM) were obtained with MEME [84] with options ‘-dna -revcomp -zoops -maxw 12.’ This produced a motif of length 12 for CTCF and HP1α and length 8 for AGO1. The PFMs (*P* <0.0005) matched at their respective summit: 15,486 (38.6%) of AGO1, 42,803 (44.4%) of CTCF and 139,176 (34.2%) of HP1α clusters.

## Additional files

Additional file 1:
**Supplementary_material.** Contains all the supplementary figures and tables. Additional file 2:
**Predicted_events_ENSG.** Supplementary data file with the predicted inclusion and skipping events. Events are provided with the coordinates of the three exons that compose the events (hg18), the regulation label (inclusion and skipping) and gene identifiers from ENSEMBL54.

## Abbreviations

5metC: 5-methycytosine
ADTree: alternate decision tree
AGO1: argonaute 1 protein
BN: Bayesian network
bp: base pair
CFS: correlation feature selection
ChIP: chromatin immunoprecipitation
CTCF: CCCTC-binding factor
H3K27me3: histone 3 lysine 27 tri-methylation
H3K36me3: histone 3 lysine 36 tri-methylation
H3K9me2: histone 3 lysine 9 de-methylation
HP1: heterochromatin protein 1
IG: information gain
RNAPII: RNA polymerase II
ROC: receiver operating characteristic
Seq: sequencing
siRNA: small interfering RNA
WSE: wrapper subset evaluator
