# Supplementary material for: A chromatin code for alternative splicing involving a putative association between CTCF and HP1α proteins
Source: BMC Biol. 2015 May 2;13:31. doi: 10.1186/s12915-015-0141-5 (PMC4446157; doi:10.1186/s12915-015-0141-5)
Supplement: Additional file 1: — Supplementary_material. Contains all the supplementary figures and tables. [file 12915_2015_141_MOESM1_ESM.pdf]

Supplementary Material

A chromatin code for alternative splicing involving a putative association between CTCF and HP1α proteins

Eneritz Agirre[1,5,*], Nicolás Bellora[1,6,*], Mariano Alló[2,7, *], Amadís Pagès[1,3], Paola Bertucci[2], Alberto R. Kornblihtt[2], Eduardo Eyras[1,4§]

[1] Universitat Pompeu Fabra. E-08003 Barcelona, Spain
[2]IFIBYNE-UBA-CONICET, Departamento de Fisiología, Biología Molecular y Celular, Facultad de Ciencias Exactas y Naturales, Universidad de Buenos Aires. (C1428EHA) Buenos Aires, Argentina.
[3]Centre for Genomic Regulation, E-08003, Barcelona, Spain
[4]Catalan Institution of Research and Advanced Studies (ICREA), E-08010 Barcelona, Spain.
[5] Present address: Institute of Human Genetics, CNRS UPR 1142, Montpellier, France.
[6] Present address: INIBIOMA, CONICET-UNComahue, Bariloche, Río Negro, Argentina.
[7] Present address: European Molecular Biology Laboratory, Meyerhofstrasse 1, 69117 Heidelberg, Germany.
*These authors contributed equally to this work
§Corresponding author

Supplementary figure 1

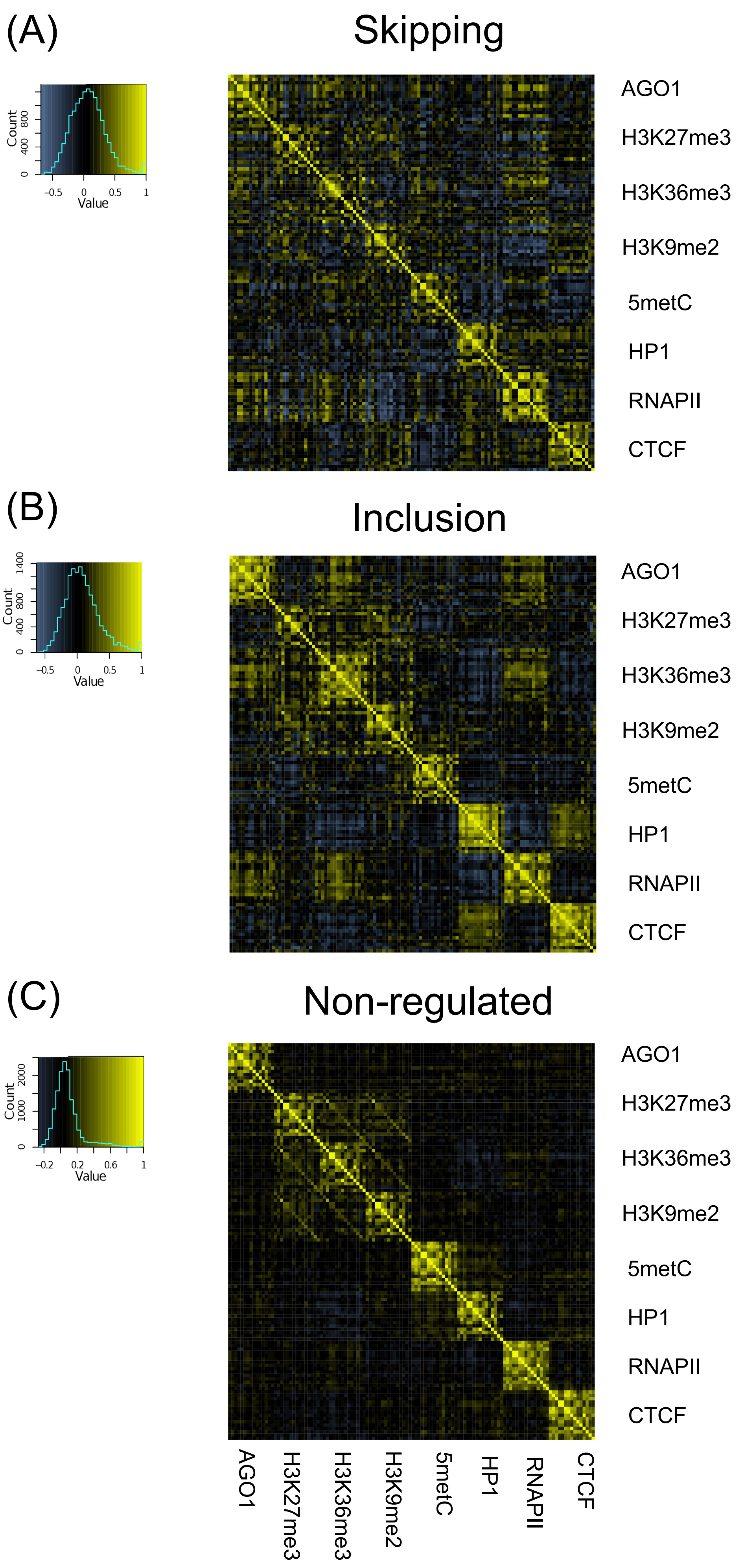

Supplementary Figure 1 - Pairwise correlation of attributes. Pairwise correlations between all attributes for skipping **(A)**, inclusion **(B)** and non-regulated **(C)** events. Each attribute is defined by an experiment – region pair. These regions are defined on exon triplets (see Figure 1A): windows flanking exons (wi1, wi2, … , wi6) are 300nt in length, junctions regions (J1, … ,J4) are 200nt in length, covering 100nt on either side of the exon boundaries. The rest of the regions were taken to be the total length of the exon (E1, E2, E3) or of the intron (I1, I2). The attributes are grouped by experiment and given in the same order in the three heatmaps. Attributes corresponding to overlapping regions show strong correlations.

Supplementary figure 2

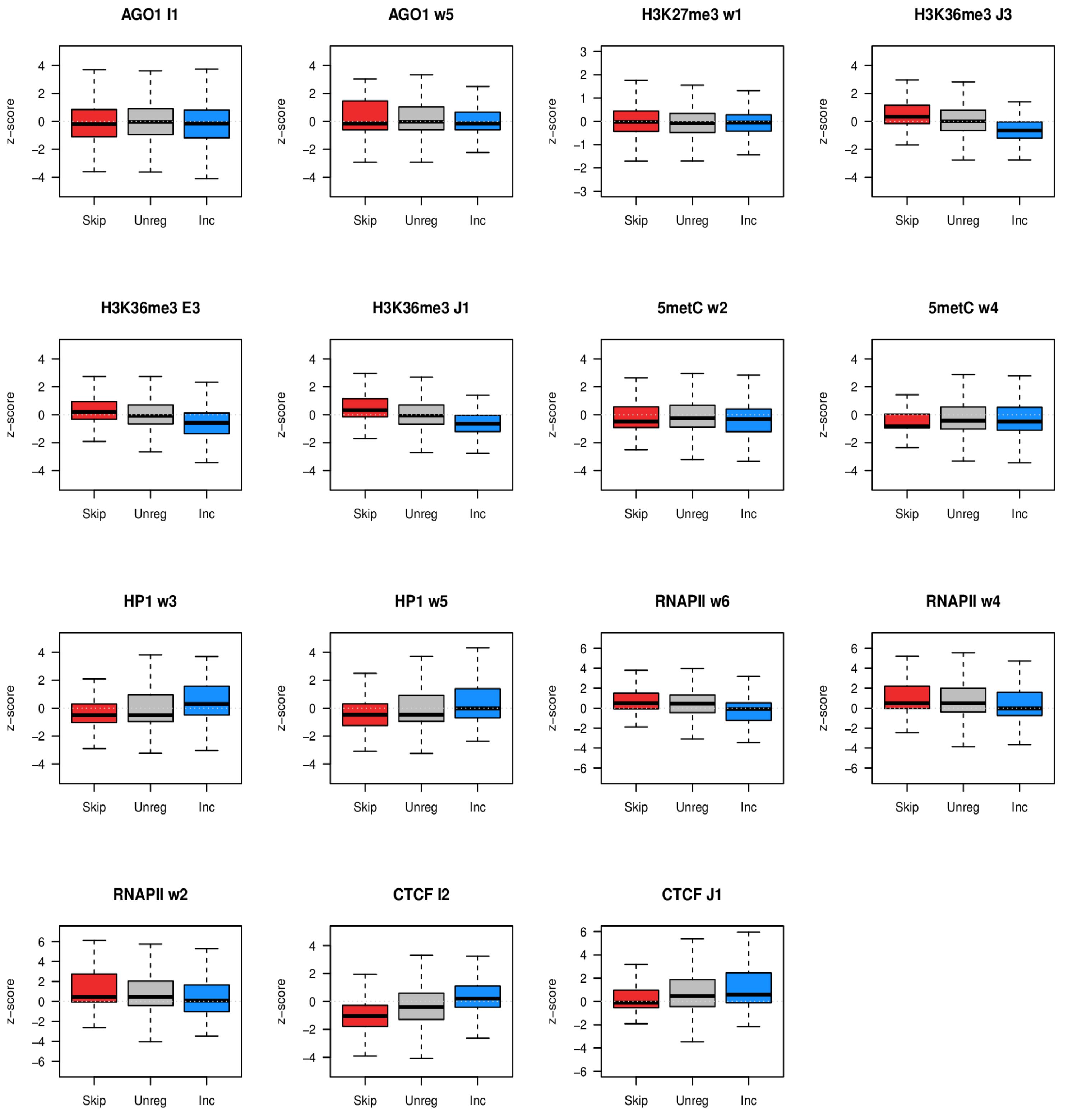

Supplementary Figure 2. Attribute distributions. Distributions of the enrichment z-scores of the correctly classified events for the 15 selected attributes compared to the set of non-regulated events. Skipping exons (red), inclusion exons (blue) and non-regulated exons (grey).

Supplementary figure 3

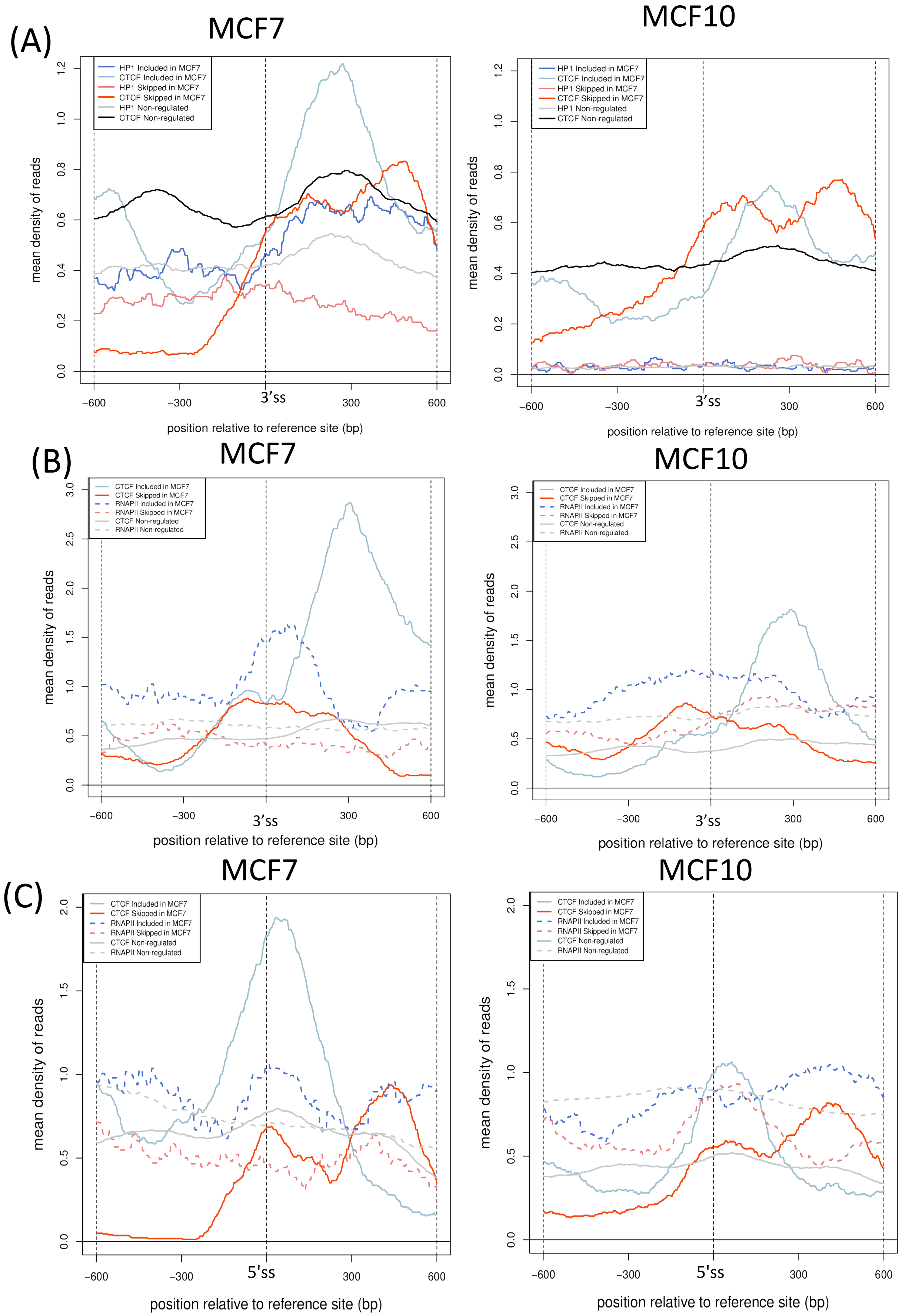

Supplementary Figure 3. Read densities for some the selected features. The profiles show the mean read densities (y-axis) from -600bp to 600bp (x-axis) centered at the 3’ss of the regulated (E2) or the 5’ss of the flanking exon E3 for both cell lines and separately for skipped exons (red), included exons (blue) and non-regulated exons (gray/black). (A) Profiles of HP1α and CTCF in MCF7 and MCF10 around 3’ splice-sites of regulated exons. (B) Profiles for the CTCF and RNAPII read densities in MCF7 and MCF10 cells around the 3’ splice-sites of the exon 3 (E3). (C) Profiles for the CTCF and RNAPII read densities in MCF7 and MCF10 cells around the 5’ splice-sites of the flanking exon E3.

Supplementary figure 4

(A)

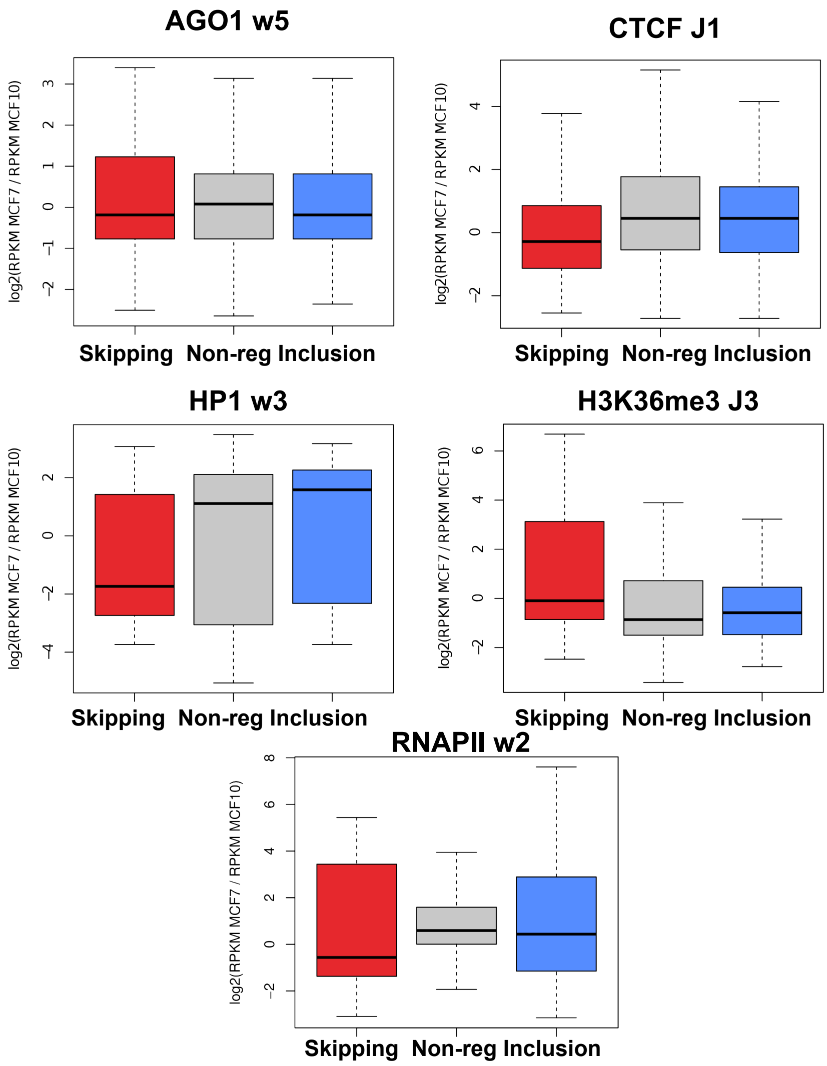

Supplementary Figure 4. Read densities for the selected features. Density differences between MCF7 and MCF10A, measured as the $\log_2$-ratio of RPKM values (y-axis) for different features characterized by an experiment-region pair, separately for skipped exons (red), inclusion exons (blue) and non-regulated exons (gray).

Supplementary Figure 5

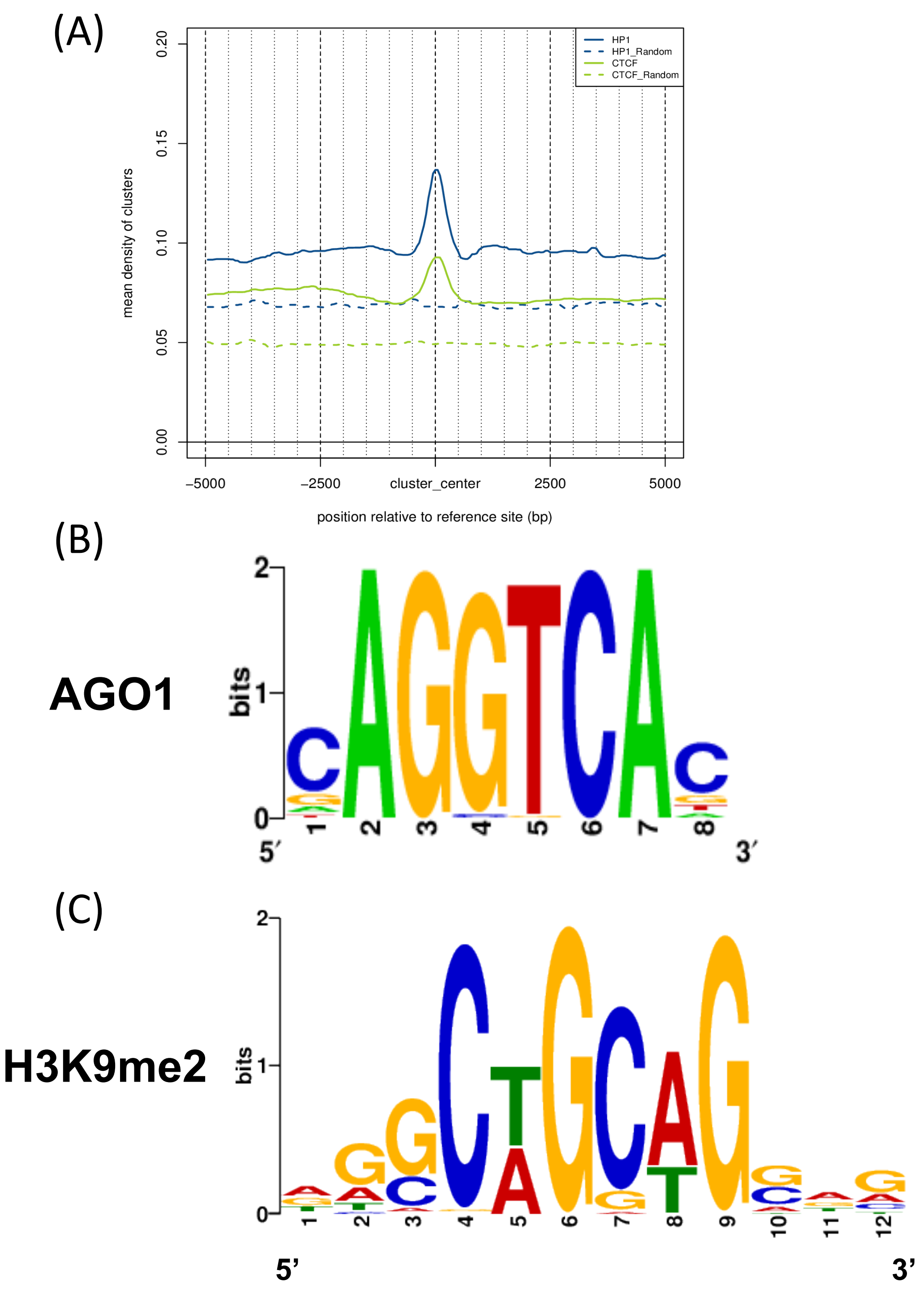

Supplementary Figure 5. (A) Mean densities of HP1 clusters centered at CTCF clusters (blue line), compared with randomized HP1 clusters (dashed blue line) in intragenic regions, 2kb away from the TSS; and mean desities of CTCF clusters (green line) centered at the HP1 clusters, compared with randomized CTCF clusters (dashed green line). Randomized clusters were calculated by relocating each cluster in an arbitrary new position in the same genic regions, avoiding the overlap with any other random clusters. **(B).** Consensus motif found for AGO1. The motif was built using the top 10 heptamers from significant AGO1 clusters and running MEME on the clusters with these motifs (see Materials and Methods). **(C).** Consensus motif found for H3K9me2 regions. The motif was built as the AGO1 motif, using the top 10 heptamers from significant H3K9me2 clusters and running MEME on the clusters with these motifs (see Materials and Methods).

Supplementary Figure 6

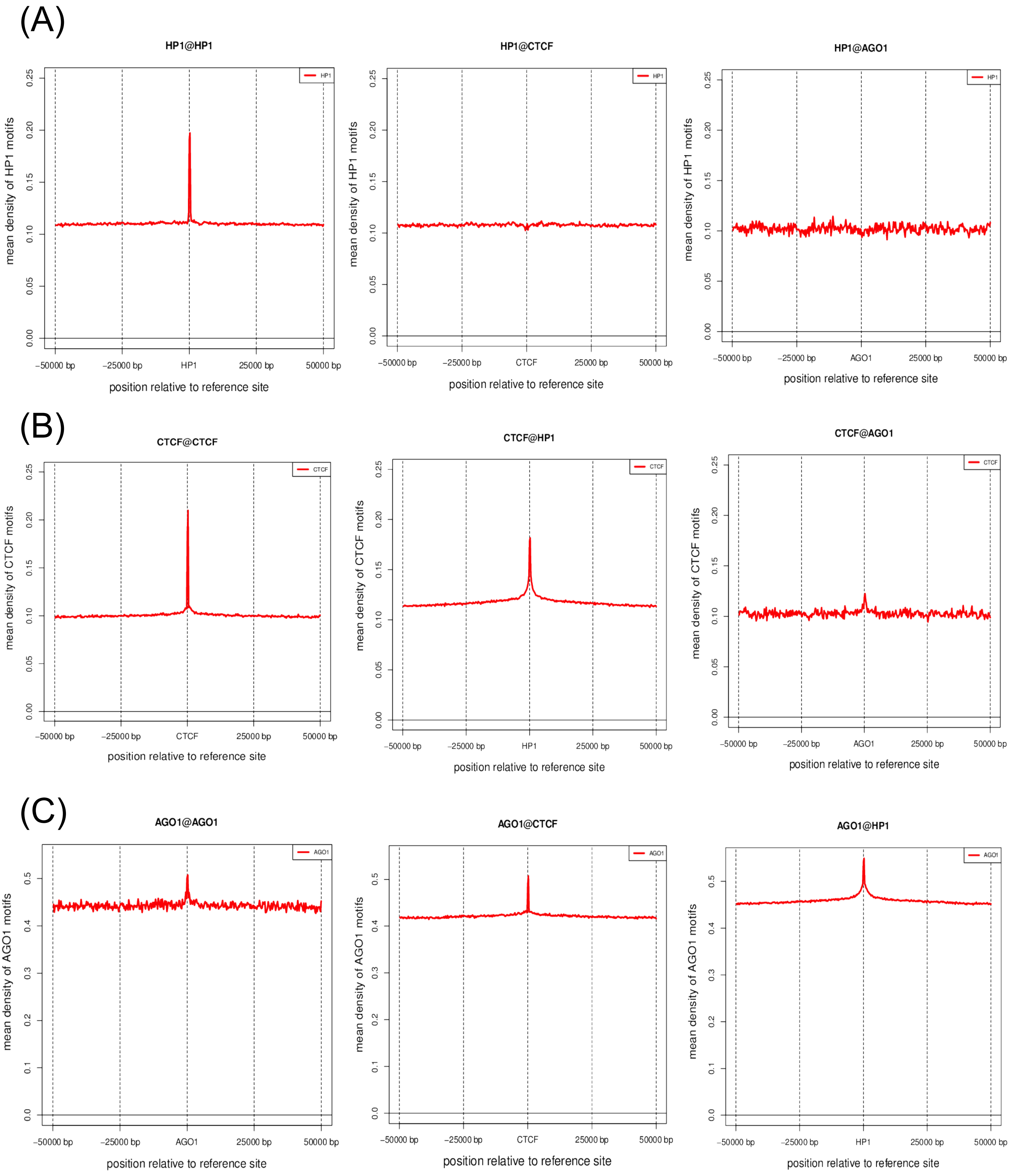

Supplementary Figure 6. Density profiles of the consensus motifs over significant clusters. Motifs were built using the top 10 heptamers from significant clusters for HP1α, CTCF and AGO1 (see Materials and Methods). Profiles for each sample show the mean density of motifs over the clusters. **(A)** HP1α, motifs **(B)** CTCF motifs and **(C)** AGO1 motifs. HP1α has the most specific motif, whereas AGO1 gives signal over the others clusters due to high level of overlap with CTCF and HP1α .

Supplementary Figure 7

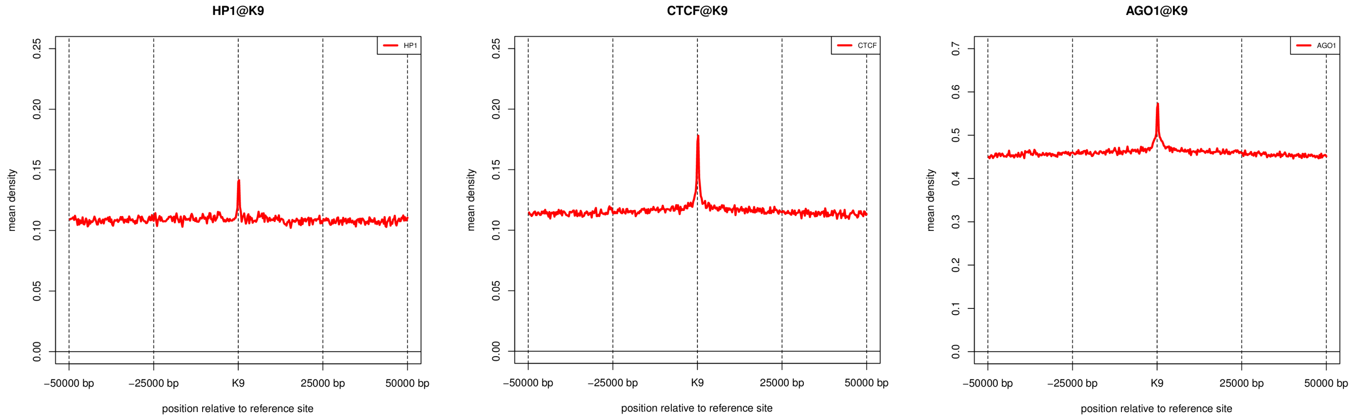

Supplementary Figure 7. Density profiles of the consensus motifs over H3K9me2 significant clusters. Motifs were built using the top 10 heptamers from significant clusters for HP1α, CTCF and AGO1 (see Materials and Methods). Profiles for each sample show the mean density of motifs over the H3K9me2

Supplementary Tables

Supplementary Table 1. Correlating features with R > 0.6 in non-regulated, events.

Non-regulated events		
Attribute	Attribute	Pearson R
H3K9me2-I1	H3K27me3-I1	0.76
H3K27me3-I2	H3K36me3-I2	0.67
H3K27me3-I2	H3K9me2-I2	0.66
H3K36me3-I1	H3K27me3-I1	0.64
H3K36me3-I1	H3K9me2-I1	0.64
H3K36me3-I2	H3K9me2-I2	0.54
H3K27me3-w2	H3K9me2-w2	0.53
H3K27me3-J1	H3K9me2-J1	0.5

Supplementary Table 2. Correlating features with R > 0.6 in inclusion events.

Inclusion events		
Attribute	Attribute	Pearson R
H3K27me3-I1	H3K9me2-I1	0.85
H3K27me3-I1	H3K36me3-I1	0.85
H3K36me3-I1	H3K9me2-I1	0.85
H3K36me3-J1	H3K9me2-J1	0.71
H3K36me3-J3	H3K9me2-J1	0.71
H3K36me3-w1	H3K9me2-J1	0.71
H3K27me3-w5	H3K9me2-w5	0.67
H3K36me3-E1	H3K9me2-J1	0.66
H3K36me3-J4	AGO1-J4	0.62
H3K36me3-J4	AGO1-J2	0.62
H3K27me3-J1	H3K9me2-I1	0.62
5metC-J1	CTCF-w5	0.61
H3K27me3-w1	H3K36me3-J1	0.61
H3K27me3-w1	H3K36me3-J3	0.61

Supplementary Table 3. Correlating features with R > 0.6 in skipping.

Skipping events		
Attribute	Attribute	Pearson R
H3K27me3-I1	H3K9me2-I1	0.81
RNAPII-w5	H3K36me3-I1	0.77
H3K27me3-I1	H3K36me3-I1	0.75
RNAPII-J2	AGO1-I2	0.74
RNAPII-I2	AGO1-w6	0.73
RNAPII-I2	AGO1-E3	0.73
H3K36me3-I1	H3K9me2-I1	0.72
RNAPII-E2	AGO1-I2	0.7
RNAPII-w3	AGO1-I2	0.69
RNAPII-I2	AGO1-J4	0.68
H3K27me3-w5	CTCF-w1	0.67
RNAPII-J3	AGO1-I2	0.67
H3K36me3-E1	RNAPII-J4	0.66
RNAPII-I1	H3K36me3-I1	0.66
H3K36me3-w1	RNAPII-J4	0.65
H3K27me3-I1	RNAPII-w2	0.65
RNAPII-J2	AGO1-J4	0.64
RNAPII-J2	AGO1-w6	0.64
H3K36me3-I1	RNAPII-I2	0.64
RNAPII-I2	AGO1-I2	0.64
RNAPII-I2	AGO1-E3	0.64
RNAPII-J2	AGO1-I2	0.64
AGO1-E3	H3K27me3-I1	0.63
AGO1-J2	H3K27me3-w2	0.63
AGO1-w6	H3K27me3-w2	0.63
H3K9me2-w2	CTCF-J3	0.63
H3K27me2-E2	H3K36me-w5	0.63
H3K36me3-J1	RNAPII-I1	0.63
H3K27me3-I1	AGO1-w6	0.63
H3K27me3-w2	AGO1-E3	0.62
RNAPII-E2	AGO1-w6	0.61
RNAPII-w2	H3K27me3-w1	0.6

Supplementary Table 4. Anticorrelating features with R <= -0.5 in inclusion events. Non-regulated events did not show any anticorrelating pair of features.

Inclusion events

Attribute	Attribute	Anticorrelation Pearson R
AGO1-J1	5metC-I1	-0.63
HP1-w1	H3K27me3-J1	-0.58
RNAPII-w5	5metC-I1	-0.58
HP1-E1	H3K27me3-J1	-0.57
CTCF-w2	H3K27me3-J1	-0.55
CTCF-w2	H3K27me3-E1	-0.55
RNAPII-w3	HP1-w3	-0.55
AGO1-w2	H3K9me2-w5	-0.52
CTCF-w2	H3K27me3-E1	-0.52
AGO1-J1	5metC-J1	-0.5

Supplementary Table 5. Anticorrelating features with R <= -0.5 in skipping events. Non-regulated events did not show any anticorrelating pair of features.

Skipping events

Attributes	Attributes	Anticorrelation R
RNAPII-w4	H3K9me2-w4	-0.68
RNAPII-w5	H3K9me2-J3	-0.67
H3K27me3-w6	RNAPII-w3	-0.64
RNAPII-I2	H3K9me2-J3	-0.63
H3K9me2-J3	RNAPII-I2	-0.63
H3K27me3-w6	RNAPII-I2	-0.63
HP1-w4	H3K9me2-J4	-0.62
RNAPII-I1	5metC-J4	-0.61
RNAPII-J3	H3K9me2-w4	-0.61
H3K27me3-J4	RNAPII-w3	-0.6
AGO1-w2	RNAPII-J1	-0.6
RNAPII-w5	H3K9me2-w4	-0.6
H3K9me2-w4	RNAPII-I2	-0.6
RNAPII-I2	H3K9me2-w4	-0.59
H3K27me3-w6	AGO1-J4	-0.59
CTCF-J1	RNAPII-w5	-0.59
RNAPII-w3	H3K9me2-w4	-0.58
H3K9me2-w4	RNAPII-w3	-0.58
RNAPII-E2	H3K9me2-w4	-0.58
H3K9me2-w4	RNAPII-w3	-0.58
H3K27me3-w3	5metC-w6	-0.57
AGO1-w2	HP1-E2	-0.56
AGO1-J1	H3K36me3-E1	-0.55
H3K27me3-I1	5metC-E3	-0.55
AGO1-J1	H3K36me3-w1	-0.55
H3K27me3-w6	AGO1-I2	-0.54

RNAPII-J2	H3K9me2-w2	-0.53
CTCF-J3	5metC-E1	-0.52
H3K9me2-I1	CTCF-J1	-0.51
CTCF-w2	H3K9me2-w2	-0.51
H3K36me3-E1	H3K9me2-w3	-0.5
AGO1-w2	RNAPII-w5	-0.5
HP1-I2	5metC-w1	-0.5
AGO1-I2	H3K36me3-w4	-0.5
HP1-I2	5metC-E1	-0.5
H3K9me2-w2	5metC-E1	-0.5

Supplementary Table 6. Scores from the three different feature selection methods for the selected attributes (indicated with an asterisk)

Attribute	WSE(%)	CFS(%)	IG (Rank)	Selected attribute
AGO1-I1	60	50	50	*
AGO1-w5	60	50	60	*
H3K36me3-J1	100	80	1	*
H3K36me3-J3	80	100	2	*
H3K36me3-I1	60	100	3	
H3K36me3-E3	90	90	6	*
H3K36me3-J4	60	60	8	
H3K27me3-w1	60	50	56	*
RNAPII-w6	90	80	5	*
RNAPII-w2	100	70	9	*
RNAPII-w4	50	80	10	*
HP1-w5	50	50	41	*
HP1-w3	50	50	54	*
5metC-w2	50	50	20	*
5metC-w4	50	50	59	*
CTCF-I2	60	90	4	*
CTCF-J1	60	90	37	*

Supplementary Table 7. Predictive performance of the model when removing all features corresponding to CTCF-I2, HP1 or AGO1 signals; or using just using a combination of them.

Correctly predicted	Only CTCF+HP1 +AGO1	Only HP1+ CTCF	Without CTCF	Without AGO1	Without HP1	Model
Total	516 (58.37%)	520(58.8%)	550(58.8%)	551(62.3%)	558(63.12%)	606(68.55%)
Inclusion	224	248	261	279	299	282
Skipping	292	272	289	272	259	324
ROC area	0.6	0.62	0.65	0.68	0.68	0.73
TPR	0.58	0.58	0.62	0.62	0.63	0.67
FPR	0.41	0.41	0.37	0.38	0.37	0.31
Precision	0.58	0.58	0.62	0.62	0.63	0.68
Recall	0.58	0.58	0.62	0.62	0.63	0.68

Supplementary Table 8. Proportion of clusters from each sample overlapping with clusters from the other samples together with the significance z-scores.

SampleA over SampleB	% of A overlapping B	zscore	Significant zscore
AGO1 over 5metC	52.6	222.83	*
AGO1 over CTCF	13.1	17.68	*
AGO1 over HP1	41.8	75.63	*
AGO1 over RNAPII	28.8	34.89	*
5metC over AGO1	3.4	-18.3	
5metC over CTCF	14.5	-15.96	
5metC over HP1	30.7	3	*
5metC over RNAPII	14.5	-20.81	
CTCF over AGO1	0.8	-13.33	
CTCF over 5metC	20.4	168.21	*
CTCF over HP1	18	112.3	*
CTCF over RNAPII	11	43.17	*
HP1 over AGO1	1.9	-14.56	
HP1 over 5metC	29.4	121.32	*
HP1 over CTCF	11.4	17.04	*
HP1 over RNAPII	10.4	-3.97	
RNAPII over AGO1	2.1	-2.62	
RNAPII over 5metC	21.2	174.28	*
RNAPII over CTCF	11.2	113.2	*
RNAPII over HP1	16.4	110.12	*
